# Iron deficiency anemia from iron malabsorption caused by proton pump inhibitors

**DOI:** 10.1002/jha2.96

**Published:** 2020-09-01

**Authors:** Michael Boxer

**Affiliations:** ^1^ Arizona Oncology Associates Tucson Arizona USA

**Keywords:** iron deficiency anemia, iron malabsorption, proton pump inhibitor

## Abstract

**Background:**

Iron deficiency anemia without evidence for blood loss can present a diagnostic challenge. Proton pump inhibitors have been associated with iron deficiency anemia for many years, yet the relationship between the two until recently was not fully understood. Treatment recommendations are lacking.

**Methods and methods:**

This study evaluated 43 iron deficient patients who were taking proton pump inhibitors, 41 of whom were unresponsive to oral iron, and for whom no etiology for the iron deficiency could be found. Two patients who had hereditary hemochromatosis never were treated with oral iron.

**Results:**

Forty‐three patients taking a proton pump inhibitor had elevated serum gastrin ≥100 pg/mL. Upon treatment with intravenous iron, 95% (41/43) responded with increased hemoglobin concentration ≥2 g/dL. Improvements were also achieved in the mean corpuscular volume, ferritin, and transferrin saturation.

**Conclusions:**

These findings suggest that proton pump inhibitors have been an under‐recognized cause for iron deficiency anemia and need to be considered in patients who are taking a proton pump inhibitor. The iron deficiency does correct with intravenous iron replacement.

## 1 INTRODUCTION

Iron deficiency anemia (IDA) is the world's most common hematologic disorder affecting over 2 billion people [1]. Not only in under‐resourced countries, but also in North America and Europe, low iron diets combined with pregnancy and menstrual loss are the cause for the vast majority of IDA [2]. Standard approaches to IDA in resource rich nations include gastrointestinal (GI) and gynecologic assessments. Often the anemia responds to oral iron replacement.

When oral iron does not lead to a significant correction of the anemia and if no source of blood loss is detected, hematologic consultation is indicated. Gastrointestinal (GI) disorders include iron malabsorptive conditions such as bariatric procedures, inflammatory bowel disease, celiac disease, *Helicobacter* infections, and autoimmune gastric atrophy. Less common hematologic conditions include paroxysmal nocturnal hemoglobinuria with associated complement‐mediated intravascular hemolysis and polycythemia rubra vera with increased iron utilization leading to deficiency.

Although proton pump inhibitors (PPI) have been associated with iron deficiency for many years, the incidence is felt to be minor [3, 4, 5]. In this study, 43 patients with iron deficiency of unclear etiology were referred to a single hematologist. After all other causes for iron deficiency were assessed, the possibility of iron malabsorption from PPI was considered.

## 2 MATERIALS AND METHODS

### 2.1 Patients

From February 2015 to December 2017, 43 patients with undiagnosed iron deficiency were referred to one hematologist at a single office or hospital in Tucson, Arizona. Sources of referral included gastroenterologists, internists, family care physicians, hospitalists, gynecologists, and nurse clinicians. No patient had documented GI blood loss, menorrhagia, gastric surgery, or bariatric procedures. All were assessed for other etiologies.

### 2.2 Diagnosis and treatment

All underwent GI assessments, which included a colonoscopy and an esophagogastroduodenoscopy (EGD) in all patients. Capsule endoscopy was performed in 13 patients. Laboratory testing for celiac disease and helicobaterial infections were performed in all patients. Serum gastrin, hemoglobin, mean corpuscular volume (MCV), ferritin, and percent transferrin saturation were measured.

Forty‐one were unresponsive to oral iron. Two who had hereditary hemochromatosis (HH) never received oral iron. Twenty‐five (61%) reported GI perturbation. Oral iron was often accompanied by stomach irritation. All received intravenous (IV) iron administered as infusions of low molecular weight (iron dextran, iron sucrose, ferric carboxymaltose, or ferumoxytol).

### 2.3 Outcomes

Response to treatment was determined by a rise in hemoglobin concentration of ≥2 g/dL. Secondary outcomes included changes in mean levels of serum ferritin, MCV, and percent transferrin saturation within 4‐6 weeks post‐treatment.

## 3 RESULTS

Of the 43 patients evaluated, 89% were women. The average serum gastrin level was 395 pg/mL (range, 114–2001 pg/mL). All had been taking a PPI. Gastric biopsies did not reveal atrophy or *Helicobacter* infections. Two with fatigue were not anemic but were iron deficient with a low MCV, transferrin saturation, and ferritin.

Overall, 79% (37/43) of patients were treated with IV iron dextran at a fixed dose of 825 mg. Ten of these 37 (27%) patients received a second infusion of IV iron dextran. Ferric carboxymaltose and ferumoxytol were administered in two divided doses in one patient each at the fixed recommended dose, 1500 and 1020 mg, respectively. Four received iron sucrose as a weight‐based dose, administered weekly for 4 weeks. One responded to ferumoxytol after a suboptimal response to iron dextran.

No patient had responded to oral iron, whereas 95% of these patients (39/41) and the two patients with hemochromatosis who never received oral iron responded to IV iron with a rise in hemoglobin concentration ≥2 g/dL (**Table I**). Increases were also seen in mean levels of ferritin, MCV, and transferrin saturation (**Table I**), with achievement of mean post‐treatment normalization.

**TABLE 1:** Laboratory measurements pre‐ and post‐treatment

	All Patients (N = 43)	
Parameters, mean (range)	Pre‐treatment	Post‐treatment
Serum gastrin (pg/mL)	395 (114‐2101)	NA
Hemoglobin (g/dL)	9.33 (6.6‐14.3)	12.9 (9.3‐16.2)
Mean corpuscular volume (MCV; fL)	76.34 (61‐93)	87.51 (69‐96)
Serum ferritin (ng/L)	12.4 (3‐73)	155 (22‐659)
Iron saturation (%)	7.15 (2‐34)	24.15 (10‐39)

Normal reference ranges: gastrin, 0‐100 pg/mL; hematocrit: 45‐52% males and 37‐48% females; MCV: 80‐100 fL; serum ferritin, 12‐300 ng/mL males and 12‐150 ng/mL females; iron saturation: 15‐50% males, 12‐45% females.

No adverse events with IV iron administration were observed. No patient discontinued IV iron.

One of the two nonresponders was obese and received one infusion of iron dextran at a non‐weight based dose of 825 mg. Both were iron deficient. Both patients experienced a rise in ferritin, MCV, and transferrin saturation. One patient had a normalization of her hemoglobin concentration with a 1.2 g/dL rise. The other patient actually experienced a small decline in hemoglobin concentration from 10.4 to 10.1 g/dL.

## 4 DISCUSSION

In 2009, 119 million prescriptions for PPI were written in the United States [6]. With the availability of over‐the‐counter esomeprazole, lansoprazole, and omeprazole, PPI usage is likely even higher. Despite a known association of PPIs with iron deficiency, the scope of their relationship is not fully understood.

In this study, 41 patients with IDA unresponsive to oral iron replacement and 2 with HH were taking a PPI and had elevated serum gastrin (≥100 pg/mL) concentrations. No other causes for IDA could be identified. All had non‐diagnostic colonoscopies and EGDs. Thirteen had a normal capsule endoscopy. Gastric and duodenal biopsies did not detect *Helicobacter* infestation, celiac disease, or gastric atrophy. Laboratory testing for celiac disease and *Helicobacter* infection was negative. No patient had gastric resections, bariatric procedures, or menorrhagia.

IV iron fully corrected the anemia in 41 of 43 treated patients. Of the two nonresponders, one was obese. One had no explanation for the lack of response. Both may have benefitted from a second iron infusion. No correlation between weight and response was observed, nor was there a clear explanation for the female predominance.

Duodenal iron absorption requires the reduction of Fe^3+^ to Fe^2+^ by the duodenal apical ferrireductase STEAP3 or the duodenal cytochrome b [7]. Dietary iron consists of both heme (32%) and non‐heme (68%) iron [7]. Heme iron (Fe^2+^) is more easily absorbed once released from globin by the activity of pancreatic enzymes. Non‐heme iron is mostly in the ferric (Fe^3+^) oxidation state and is less readily absorbed. Non‐heme iron is bound to cereals, vegetables, and beans, which require the acidic gastric environment to increase its bioavailability. PPI limits hydrogen ion (H^+^) production by inhibiting the hydrogen/potassium pump within gastric parietal cells. Subsequently, less Fe^3+^ becomes available for intestinal reduction and absorption. The increased gastric pH leads to G cell hyperplasia with an increase in serum gastrin. With less ferric iron available for reduction, less ferrous iron is absorbed, and iron deficiency develops.

After bariatric surgery, ingested iron no longer can be efficiently conjugated to vitamin C, amino acids, and sugars in the presence of gastric acid, which leaves ferric hydroxide in the unabsorbable oxidation state [8].

The ability of PPI to reduce iron absorption has been recognized for over a decade and has been exploited for the treatment of HH, a condition associated with excess iron absorption due to a mutation in the HFE gene. HH is associated with damage to numerous organs such as the heart, pancreas, and liver due to the release of toxic free oxygen radicals from unbound ferric ions [9, 10]. In 2007, Hutchinson et al. demonstrated an 80% reduction in blood removed in patients with HH who were taking a PPI [11]. More recently, Vanclooster et al. demonstrated significantly fewer phlebotomies in HH patients who were taking a PPI in a double‐blind randomized placebo‐controlled trial [12]. Neither study reported iron deficiency from PPI.

Two patients in this study did have HH. Each had homozygosity for the C282Y mutation. Neither took oral iron. Both continue to take PPI. One has only been phlebotomized once in April 2005, and the other twice and as recently as May 2019. Each was infused once with IV iron, which raised their ferritin to 33 and 55 ng/mL, respectively.

Iron overload within the liver does occur in both non‐ and minimally transfused hereditary anemia characterized by chronic hemolysis or inefficient erythropoiesis. The anemia lowers hepcidin and subsequently increased iron absorption. PPI effectiveness in decreasing liver iron concentration is under investigation [13].

Iron deficiency from PPI has been recognized for many years but is usually an infrequent event in published series. The first case report was in 2002 that described IDA induced by long‐term usage of a PPI [14]. In a 2004 case report, Sharma et al described two patients who required a PPI for severe gastritis and developed iron deficiency that did not respond to oral iron until the PPI was discontinued [15]. If continuation of the PPI were necessary, they suggested that IV iron be considered [15]. In a retrospective study, Sarzynski et al reported that 98 patients who were receiving PPI for >1 year developed significant decreases in the hematocrit, hemoglobin concentration, and MCV, compared with matched controls [6]. More than 20% had a significant decrease in hemoglobin concetnration (≥1 g/dL). But given the retrospective nature of this electronic medical record chart review, no treatment for the iron deficiency was discussed [6]. A 2014 case report from Japan described the likely association between iron deficiency and a PPI. The anemia did not correct with oral iron therapy until the PPI was discontinued and an H2 blocker substituted [16]. Also in Japan, Imai et al in 2018 described one patient who developed iron deficiency after taking a PPI for 25 years [17]. Also from Japan in cardiac patients, use of PPI was associated with anemia in 51%, compared to 19% in those not receiving a PPI (*P* < .001). No conclusions were drawn, other than that long‐term PPI users may develop IDA. This type of anemia was not delineated [18].

In another study, PPI was used to control hydrogen ion secretion in patients with Zollinger‐Ellison Syndrome. PPI treatment led to reduction in serum gastrin but did not cause iron deficiency. Following a curative gastric resection, 89 patients were taking omeprazole, nine were taking histamine H2‐antagonists, and 11 were taking no medication. Mean duration of PPI was 5.7 years (range, 0.7‐12.5) [19]. Acid hyposecretion occurred in 45% of patients. There were no significant differences between patients with or without acid hyposecretion, taking or not taking omeprazole, having different durations of omeprazole treatment, for any iron parameter, hematologic parameter, or for the frequency of iron deficiency. Males and females did not differ in percentage with low ferritin levels or percentage with iron deficiency [19].

In a recent Dutch study, in which full (ie, continuous for >1 year) and limited (intermittent use over an undefined time period) users of PPIs had an adjusted odds ratio for developing iron deficiency of 3.60 for individuals taking PPI compared to those who did not [20]. This is an extremely important study that finally proved that PPI can cause IDA. By using a huge United Kingdom database, 26,806 iron deficient patients were compared to a similar number of controls. However, as this was a retrospective chart study no treatment recommendations were possible. Interestingly, 75.3% of the patients were female. A Kaiser Permanente study showed a similar odds ratio for iron deficiency associated with 2 years of PPI use [21]. These studies concluded that chronic PPI use increases the risk for iron deficiency. Assessment for iron deficiency should be considered in those being treated with chronic PPI usage.

Our study demonstrates that IV iron corrects the iron deficiency caused by iron malabsorption from PPIs, which had not corrected with oral iron. Our study complements the Dutch study, with the logical conclusion that intravenous iron would be considerably more effective than oral iron for an iron malabsorptive state.

In an editorial involving the Dutch and Kaiser Permanente studies, McCarthy agreed that PPI can cause iron deficiency and recognized that treatment of iron deficiency has been poorly studied when continuous need for PPI is present. He concluded that IV iron therapy should be avoided [22].

Three studies have assessed the association of long‐term PPI therapy with bone fractures, calcium homeostasis, B12, iron, magnesium, and other vitamin and mineral deficiencies. All concluded that PPI will limit iron absorption but concluded that this effect needs more assessment and no recommendations were made. Interestingly, two of the studies focused more on vitamin B12 malabsorption than iron malabsorption.

## 5 CONCLUSIONS

IDA or iron depletion with anemia due to iron malabsorption from PPI use is much more common than previously recognized and should be considered for an iron deficiency patient without evidence for other causes for iron deficiency. Treatment with IV iron is much more effective than oral iron, associated with fewer adverse events, and should be considered as frontline therapy.

## DISCLOSURES

Dr. Boxer has participated in speaker bureaus for Rigel, Incyte, and AbbVie.

## References

[1] Camaschella C. Iron‐deficiency anemia. N Engl J Med. 2015;372(19):1832–1843. 25946282 10.1056/NEJMra1401038

[2] Kassebaum NJ , Jasrasaria R , Naghavi M , Wulf SK , Johns N , Lozano R , et al. A systematic analysis of global anemia burden from 1990 to 2010. Blood 2014;123(5):615–624. 24297872 10.1182/blood-2013-06-508325 PMC3907750

[3] Heidelbaugh JJ. Proton pump inhibitors and risk of vitamin and mineral deficiency: evidence and clinical implications. Ther Adv drug Saf. 2013;4(3):125–133. 25083257 10.1177/2042098613482484 PMC4110863

[4] Johnson DA , Oldfield EC 4th . Reported side effects and complications of long‐term proton pump inhibitor use: dissecting the evidence. Clin Gastroenterol Hepatol. 2013;11(5):458. 23247326 10.1016/j.cgh.2012.11.031

[5] Ito T , Jensen RT. Association of long‐term proton pump inhibitor therapy with bone fractures and effects on absorption of calcium, vitamin B12, iron, and magnesium. Curr Gastroenterol Rep. 2010;12(6):448–457. 20882439 10.1007/s11894-010-0141-0 PMC2974811

[6] Sarzynski E , Puttarajappa C , Xie Y , Grover M , Laird‐Fick H , Association between proton pump inhibitor use and anemia: a retrospective cohort study. Dig Dis Sci. 2011;56(8):2349–2353. 21318590 10.1007/s10620-011-1589-y

[7] Andrews NC. Forging a field: the golden age of iron biology. Blood. 2008;112(2):219–230. 18606887 10.1182/blood-2007-12-077388 PMC2442739

[8] Auerbach M. New paradigms and therapies for iron replacement in iron deficiency anemia. Clin Adv Hematol Oncol. 2018;16(11):712–715. 30543585

[9] Bomford A. Genetics of haemochromatosis. Lancet (London, England) 2002;360(9346):1673–1681. 10.1016/S0140-6736(02)11607-2 12457803

[10] Pietrangelo A. Hereditary hemochromatosis–a new look at an old disease. N Engl J Med. 2004;350(23):2383–2397. 15175440 10.1056/NEJMra031573

[11] Hutchinson C , Geissler CA , Powell JJ , Bomford A . Proton pump inhibitors suppress absorption of dietary non‐haem iron in hereditary haemochromatosis. Gut. 2007;56(9):1291–1295. 17344278 10.1136/gut.2006.108613 PMC1954964

[12] Vanclooster A , van Deursen C , Jaspers R , Cassiman D , Koek G , Proton Pump Inhibitors Decrease Phlebotomy Need in HFE Hemochromatosis: Double‐Blind Randomized Placebo‐Controlled Trial. Gastroenterology 2017;153(3):678‐680.e2. 28624580 10.1053/j.gastro.2017.06.006

[13] Van Vuren AJ , Biemond BJ , Kerkhoffs J‐L , Schols SEM , Rijneveld AW , Nur E , et al. Proton Pump Inhibition for Secondary Hemochromatosis in Hereditary Anemia, a Phase III Placebo Controlled Randomized Cross‐over Trial in Progress. Blood 2019;134(Suppl 1):960.31395603

[14] Khatib MA , Rahim O , Kania R , Molloy P . Iron deficiency anemia: induced by long‐term ingestion of omeprazole. Dig Dis Sci. 2002;47(11):2596–2597. 12452401 10.1023/a:1020540916482

[15] Sharma VR , Brannon MA , Carloss EA . Effect of omeprazole on oral iron replacement in patients with iron deficiency anemia. South Med J. 2004;97(9):887–889. 15455980 10.1097/01.SMJ.0000110405.63179.69

[16] Hashimoto R , Matsuda T , Chonan A . Iron‐deficiency anemia caused by a proton pump inhibitor. Intern Med. 2014;53(20):2297–2299. 25318791 10.2169/internalmedicine.53.2743

[17] Imai R , Higuchi T , Morimoto M , Koyamada R , Okada S , Iron Deficiency Anemia Due to the Long‐term Use of a Proton Pump Inhibitor. Intern Med. 2018;57(6):899–901. 29151538 10.2169/internalmedicine.9554-17 PMC5891535

[18] Shikata T , Sasaki N , Ueda M , Kimura T , Itohara K , Sugahara M , et al. Use of proton pump inhibitors is associated with anemia in cardiovascular outpatients. Circ J. 2015;79(1):193–200. 25392070 10.1253/circj.CJ-14-0582

[19] Stewart CA , Termanini B , Sutliff VE , Serrano J , Yu F , Gibril F , et al. Iron absorption in patients with Zollinger‐Ellison syndrome treated with long‐term gastric acid antisecretory therapy. Aliment Pharmacol Ther. 1998;12(1):83–98. 10.1046/j.1365-2036.1998.00274.x 9692706

[20] Tran‐Duy A , Connell NJ , Vanmolkot FH , Souverein PC , de Wit NJ , Stehouwer CDA , et al. Use of proton pump inhibitors and risk of iron deficiency: a population‐based case‐control study. J Intern Med. 2019;285(2):205–214. 30141278 10.1111/joim.12826

[21] Lam JR , Schneider JL , Quesenberry CP , Corley DA . Proton Pump Inhibitor and Histamine‐2 Receptor Antagonist Use and Iron Deficiency. Gastroenterology 2017;152(4):821‐829.e1. 27890768 10.1053/j.gastro.2016.11.023

[22] McCarthy D . Iron deficiency anaemia due to proton pump inhibitors: clinical impact revealed. J Intern Med. 2019;285(2):245–247. 30357965 10.1111/joim.12846

